# Nitrogen-Doped Graphene Uniformly Loaded with Large Interlayer Spacing MoS_2_ Nanoflowers for Enhanced Lithium–Sulfur Battery Performance

**DOI:** 10.3390/molecules29204968

**Published:** 2024-10-21

**Authors:** Zhen Wu, Wenfeng He, Renjie Xie, Xuan Xiong, Zihan Wang, Lei Zhou, Fen Qiao, Junfeng Wang, Yan Zhou, Xinlei Wang, Jiajia Yuan, Tian Tang, Chenyao Hu, Wei Tong, Lubin Ni, Xin Wang, Yongsheng Fu

**Affiliations:** 1 School of Energy and Power Engineering, Jiangsu University, Zhenjiang 212013, China; 18719810161@163.com (R.X.); 19709101790@163.com (X.X.); l.zhou@ujs.edu.cn (L.Z.); wangxinlei@ujs.edu.cn (X.W.); 2 Key Laboratory for Soft Chemistry and Functional Materials of Ministry of Education, Nanjing University of Science and Technology, Nanjing 210094, Chinajiajiayuan@njust.edu.cn (J.Y.);; 3 FEB Research Institute, Far East Battery, Wuxi 214200, China; 4 College of Science & Institute of Materials Physics and Chemistry, Nanjing Forestry University, Nanjing 210037, China; 5 School of Mechanical and Aerospace Engineering, Nanyang Technological University, 50 Nanyang Avenue, Singapore 639798, Singapore; tomson90@126.com; 6 School of Chemistry and Chemical Engineering, Yangzhou University, Yangzhou 225002, China; lbni@yzu.edu.cn

**Keywords:** lithium–sulfur batteries, electrocatalysts, MoS_2_ nanoflowers, interlayer spacing, catalytic conversion

## Abstract

Lithium–sulfur (Li-S) batteries offer a high theoretical energy density but suffer from poor cycling stability and polysulfide shuttling, which limits their practical application. To address these challenges, we developed a PANI-modified MoS_2_-NG composite, where MoS_2_ nanoflowers were uniformly grown on graphene oxide (GO) through PANI modification, resulting in an increased interlayer spacing of MoS_2_. This expanded spacing exposed more active sites, enhancing polysulfide adsorption and catalytic conversion. The composite was used to prepare MoS_2_-NG/PP separators for Li-S batteries, which achieved a high specific capacity of 714 mAh g^−1^ at a 3 C rate and maintained a low capacity decay rate of 0.085% per cycle after 500 cycles at 0.5 C. The larger MoS_2_ interlayer spacing was key to improving redox reaction kinetics and suppressing the shuttle effect, making the MoS_2_-NG composite a promising material for enhancing the performance and stability of Li-S batteries.

## 1. Introduction

The quest for high-energy-density storage systems is driving significant advancements in battery technologies, with lithium–sulfur (Li-S) batteries emerging as one of the most promising candidates. Li-S batteries offer a theoretical energy density of 2600 Wh kg^−1^ and a specific capacity of 1675 mAh g^−1^, making them highly attractive for applications in electric vehicles, portable electronics, and large-scale energy storage systems [1,2,3]. These advantages are primarily attributed to the high energy density of sulfur and its abundance, low cost, and environmental benignity. However, despite these promising attributes, the practical application of Li-S batteries faces several critical challenges that have hindered their commercialization. One of the major obstacles in Li-S batteries is the inherent insulating nature of sulfur and its discharge products, lithium sulfide (Li_2_S), which leads to poor electrical conductivity and limits the rate capability of the battery [4,5,6]. Furthermore, the dissolution of intermediate lithium polysulfides (LiPSs) into the electrolyte during the charge–discharge process leads to the infamous shuttle effect. This shuttle effect, where LiPSs migrate between the cathode and anode, results in active material loss, severe capacity fading, and poor Coulombic efficiency [7,8]. Additionally, the substantial volume expansion (up to 78%) of sulfur during lithiation further exacerbates the mechanical instability of the cathode, leading to rapid performance degradation over repeated cycles [9].

To overcome these challenges, researchers have focused on various strategies to enhance the performance of Li-S batteries. One of the most effective approaches is the incorporation of catalysts that can facilitate the redox reactions of LiPSs and suppress the shuttle effect. Catalysts play a crucial role in improving the kinetics of the LiPS conversion reactions, stabilizing the intermediate species, and enhancing the overall electrochemical performance of the battery [10,11,12]. Among various catalysts, high-polarity transition metal compounds, such as metal oxides [13,14], nitrides [15,16], sulfides [17,18], and carbides [19,20], have been widely used due to their strong chemical adsorption and catalytic effects on LiPS conversion. Molybdenum disulfide (MoS_2_), a transition metal sulfide, stands out due to its diverse structure, high catalytic activity, electrochemical stability, and strong affinity for polysulfides, making it a promising candidate for improving the performance of sulfur cathodes [21]. Compared to other electrode materials, MoS_2_ offers an optimal combination of chemical properties and structural versatility that enhances the redox reaction kinetics and suppresses the shuttle effect [22]. However, despite its potential, MoS_2_ suffers from certain limitations, such as poor electrical conductivity and limited interlayer spacing, which can lead to aggregation and reduced catalytic activity [23]. These issues hinder the full utilization of MoS_2_ as an effective catalyst in Li-S batteries.

To address these limitations, researchers have explored the integration of MoS_2_ with conductive carbon-based materials such as graphene [24]. Graphene, with its exceptional electrical conductivity, large surface area, and mechanical flexibility [25], serves as an ideal support for MoS_2_, improving its dispersion and structural stability. To further enhance the catalytic performance of MoS_2_ in lithium–sulfur batteries, several modification strategies have been developed. These include engineering the structure of MoS_2_, such as creating defect-rich or ultra-thin layers to expose more catalytic sites [26,27], and doping with heteroatoms like nitrogen to improve conductivity and interaction with LiPSs [28]. Nitrogen doping, in particular, has been shown to modify the electronic structure of MoS_2_, reducing the energy barrier for LiPS adsorption and accelerating the redox reaction kinetics [29]. Furthermore, expanding the interlayer spacing of MoS_2_ further increases active site accessibility and facilitates LiPS diffusion [30]. Additionally, coupling MoS_2_ with other materials in heterostructures can create multifunctional catalytic systems that improve overall battery performance [31]. These strategies greatly enhance the effectiveness of MoS_2_ in Li-S batteries.

In our study, we report the synthesis of nitrogen-doped graphene-supported MoS_2_ nanoflowers (MoS_2_-NG) through a novel approach that involves the uniform growth of MoS_2_ nanoflowers on graphene followed by high-temperature calcination to achieve nitrogen doping. The strong interaction between MoS_2_ and graphene in the MoS_2_-NG composite ensures structural integrity and stability during cycling. Moreover, the expanded interlayer spacing of MoS_2_ nanoflowers exposes a greater number of in-plane active sites, which not only enhances the chemical adsorption of LiPSs but also catalyzes their conversion, thus mitigating the shuttle effect. As a result, the MoS_2_-NG composite demonstrates significantly improved electrochemical performance, including enhanced cycling stability, higher Coulombic efficiency, and superior rate capability. This innovative approach offers a promising pathway for the practical application of Li-S batteries and contributes to the ongoing development of advanced energy storage technologies.

## 2. Results and Discussion

### 2.1. Morphology and Structure of Materials

Firstly, the microstructure of GO and PANI-GO was characterized using scanning electron microscopy (SEM). Figure S1a–c displays the SEM images of GO at different magnifications, revealing a layered structure with large sheet dimensions and no apparent aggregation. After the PANI treatment, as shown in Figure S1d–f, the sheet size of GO reduced and numerous wrinkles appeared. The elemental distribution of PANI-GO, illustrated in Figure S1g–i, indicates the presence of C, O, and N elements. The O element originates from the oxygen-containing functional groups on the GO surface, while the presence of N confirms that PANI has been successfully and uniformly coated onto the GO surface. Subsequently, both GO and PANI-GO were dispersed into solutions, and their Zeta potentials were measured. As shown in Figure S2, the initial Zeta potential of GO was −67.3 mV. Given that Mo_7_O_24_^6−^ is negatively charged, electrostatic repulsion between GO and Mo_7_O_24_^6−^ hinders the adsorption of Mo_7_O_24_^6−^ on the GO surface and the uniform growth of MoS_2_. After modification with PANI, the Zeta potential shifted to 31.8 mV. Under these conditions, Mo_7_O_24_^6−^ is uniformly and strongly adsorbed onto the PANI-GO surface via electrostatic attraction and chelation, ensuring the uniform distribution of MoS_2_ on the GO surface without aggregation.

**Figure 1:**
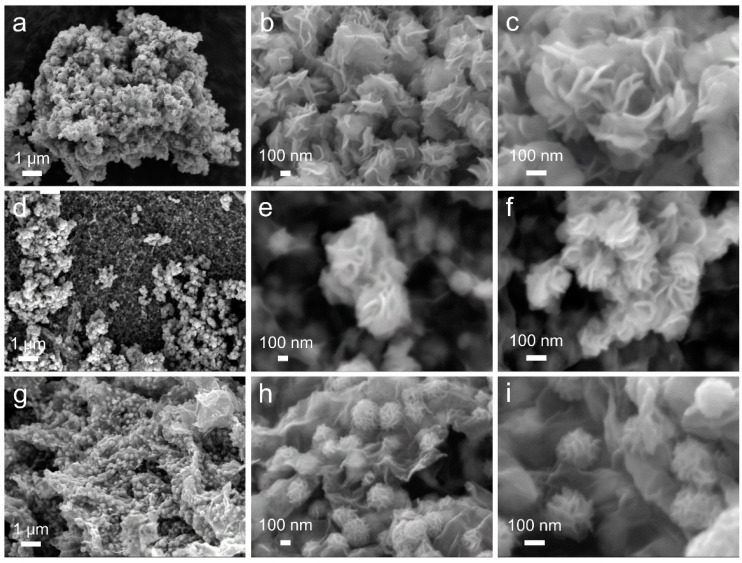
SEM images of MoS_2_ (**a**–**c**), MoS_2_-G (**d**–**f**), and MoS_2_-NG (**g**–**i**) at different magnifications.

Figure 1a–c presents the SEM images of MoS_2_ at different magnifications, where the morphology of MoS_2_ is observed as nanosheet-assembled nanoflowers, with diameters ranging from approximately 200 to 300 nm and sheet thicknesses of around 10 nm. Due to the absence of supporting material, pure MoS_2_ nanoflowers are not uniformly dispersed and exhibit slight aggregation. Figure 1d–f depicts the SEM images of MoS_2_-G, where GO was not modified with PANI. Even though some MoS_2_ nanoflowers successfully grew uniformly on the GO surface, a significant portion of MoS_2_ nanoflowers still heavily agglomerated on the GO surface. Upon increasing the magnification of the SEM images, it is evident that the morphology of the MoS_2_ nanoflowers in MoS_2_-G does not significantly differ from that of pure MoS_2_, and aggregation remains prominent.

In contrast, Figure 1g–i displays the SEM images of MoS_2_-NG at different magnifications. Due to the PANI modification, MoS_2_ nanoflowers are uniformly distributed on the GO surface without noticeable aggregation, maintaining a diameter of approximately 200–300 nm. Comparing the microstructures of MoS_2_, MoS_2_-G, and MoS_2_-NG under SEM, it can be inferred that the PANI modification effectively changed the surface charge of GO from negative to positive. This surface modification created sites capable of uniformly adsorbing Mo_7_O_24_^6−^, effectively preventing the aggregation of MoS_2_ nanoflowers during their formation and promoting the growth of thinner nanosheets. Compared to pure MoS_2_ and MoS_2_-G, MoS_2_-NG exhibits more exposed active sites, offering superior catalytic activity for polysulfides when applied to the modification of separators in lithium–sulfur batteries.

**Figure 2:**
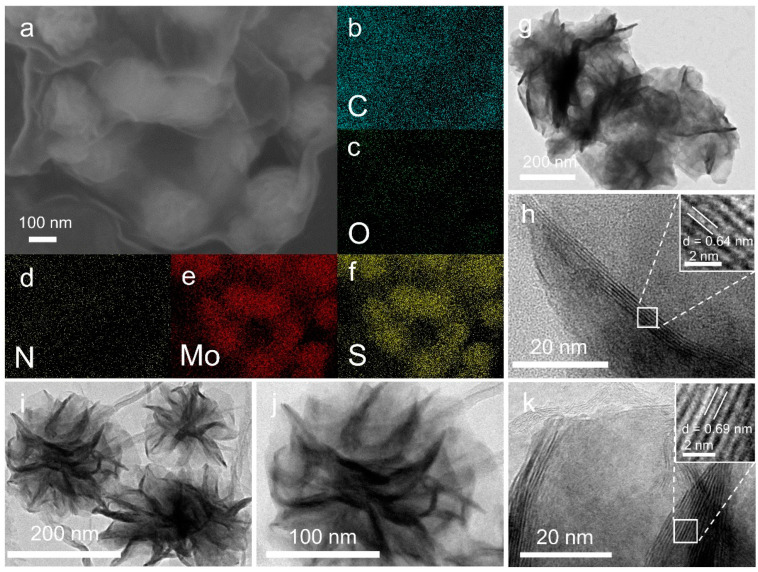
SEM images (**a**) and element distribution diagrams (**b**–**f**) of MoS_2_-NG (C, O, N, Mo, S elements); TEM images of MoS_2_ (**g**,**h**) and MoS_2_-NG (**i**–**k**) at different magnifications.

Figure 2a–f presents the elemental distribution of MoS_2_-NG, which primarily consists of C, O, N, Mo, and S elements. The C, O, and N elements originate from the GO, while the Mo and S elements are derived from the MoS_2_ nanoflowers. Transmission electron microscopy (TEM) images of MoS_2_ (Figure 2g,h) reveal significant stacking of the nanosheets and aggregation among the nanoflowers. Fourier transform calculations show an interlayer spacing of 0.64 nm, corresponding to the (002) plane of MoS_2_. In contrast, as shown in Figure 2i–k, the morphology of MoS_2_ nanoflowers in MoS_2_-NG is more uniform and well-distributed on the GO surface without noticeable aggregation. Additionally, the interlayer spacing of the (002) plane in MoS_2_-NG is measured at 0.69 nm, larger than that of pure MoS_2_. This indicates that the PANI-modified GO not only ensures the uniform growth of MoS_2_ nanoflowers but also increases the interlayer spacing, enriching the in-plane active sites, which is beneficial for the adsorption and catalytic conversion of polysulfides [32], thereby enhancing the electrochemical performance of lithium–sulfur batteries.

The catalyst material was loaded onto the commercial PP membrane surface using a simple vacuum filtration method. To further observe the surface morphology and the thickness of the modified membrane, scanning electron microscopy (SEM) was used to capture images of the top surface and cross-section of the PP, MoS_2_/PP, MoS_2_-G/PP, and MoS_2_-NG/PP membranes. Figure S3a,b shows the SEM images of the commercial PP membrane at different magnifications, where numerous pores are visible on the surface. These pores facilitate electrolyte infiltration and lithium-ion transport. However, these same pores also allow the migration of soluble polysulfide intermediates to the anode during the charge/discharge cycles of the lithium–sulfur battery, contributing to the shuttle effect.

Figure S3c shows the cross-sectional SEM image of the PP membrane, with a thickness of approximately 25 μm. Since no catalyst material is loaded, the surface appears smooth at low magnifications. Figure S3d,e displays the SEM images of the MoS_2_/PP membrane surface, where the MoS_2_ nanoflowers are well-structured and almost fully cover the membrane surface. However, due to significant stacking and aggregation of the MoS_2_ nanoflowers, the modified membrane surface is not entirely smooth. This unevenness is also evident in the cross-sectional SEM image of the MoS_2_/PP membrane (Figure S3f), where the modification layer thickness is approximately 3.40 μm, showing uneven loading across different areas of the membrane, leading to variations in the catalyst loading. In comparison, the surface of the MoS_2_-G/PP membrane is relatively smoother, as shown in Figure S3g,h. The MoS_2_-G also fully covers the PP membrane surface, obscuring the original pore structure of the membrane. However, due to the absence of PANI modification on the GO surface, the MoS_2_ nanoflowers do not grow uniformly on the GO, leading to localized aggregation. Figure S3i shows the cross-sectional SEM image of the MoS_2_-G/PP membrane, where the structure is more porous compared to pure MoS_2_, with a modification layer thickness of approximately 4.68 μm.

A comparative analysis of the SEM images of the MoS_2_/PP and MoS_2_-G/PP membranes reveals a common issue: although the catalyst material can completely cover the PP membrane pores, physically blocking polysulfides, the aggregation of MoS_2_ nanoflowers obscures numerous catalytic active sites and results in uneven distribution of these sites across the membrane surface. This uneven distribution leads to variations in catalytic efficiency across different areas of the membrane, thereby affecting the overall stability of the redox kinetics in the battery system. Figure S3j,k shows the SEM images of the MoS_2_-NG/PP membrane surface at different magnifications. Unlike the previous cases, it is evident that the PANI modification altered the surface charge of the GO, allowing the MoS_2_ nanoflowers to grow uniformly on the GO surface. When MoS_2_-NG is loaded onto the PP membrane, no significant aggregation is observed, and the overall surface morphology is very smooth. As shown in Figure S3l, the modification layer thickness of the MoS_2_-NG/PP membrane is approximately 11.49 μm. This increased thickness is attributed to the uniform loading of MoS_2_ nanoflowers on the GO, resulting in a more porous structure with a larger specific surface area. Consequently, under the same catalyst loading per unit area, the modification layer is thicker. Since the MoS_2_ nanoflowers do not aggregate, MoS_2_-NG effectively combines physical blocking and chemical adsorption of polysulfides, exposing numerous catalytic active sites and optimizing the kinetics of lithium–sulfur battery reactions, accelerating the conversion of polysulfides to the final product (Li_2_S).

**Figure 3:**
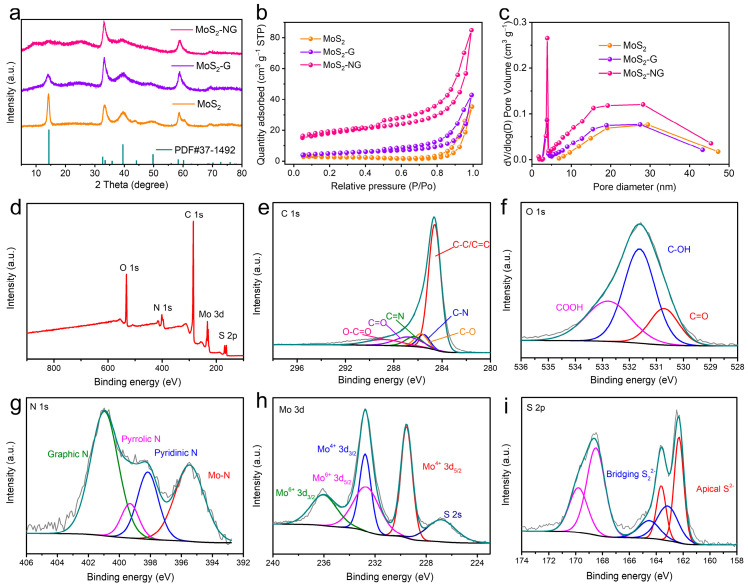
(**a**) XRD patterns of MoS_2_, MoS_2_-G, and MoS_2_-NG. (**b**) Pore size distributions (**c**) of MoS_2_, MoS_2_-G, and MoS_2_-NG. XPS full scan of MoS_2_-NG (**d**) and high-resolution XPS spectra of C 1s (**e**), O 1s (**f**), N 1s (**g**), Mo 3d (**h**), and S 2p (**i**).

The XRD patterns of the samples are shown in Figure 3a. By comparing with the standard card (JCPDS No. 37-1492), the peaks at approximately 14.4°, 33.5°, and 58.9° in MoS_2_, MoS_2_-G, and MoS_2_-NG correspond to the (002), (100), and (110) crystal planes of MoS_2_, respectively. However, in the XRD pattern of MoS_2_-NG, the intensity of the (002) peak is weakened, and a weaker, broad peak appears around 10°. According to Bragg’s equation, this suggests that the layered structure of MoS_2_ in MoS_2_-NG is disordered and has an increased interlayer spacing. This structural feature is advantageous for exposing more in-plane active sites, which can facilitate the catalytic conversion of polysulfides. In the Raman spectra (Figure S4), compared to MoS_2_ and MoS_2_-G, MoS_2_-NG exhibits lower intensity for the E_2g_^1^ and A_1g_ characteristic peaks. This reduction in intensity can be attributed to the structural disorder introduced by nitrogen doping, which weakens the vibrational modes of the MoS_2_ layers. Moreover, the A_1g_ peak in MoS_2_-NG shifts to lower wavenumbers, which is typically associated with a reduction in the number of MoS_2_ layers [33]. The nitrogen doping and uniform distribution of MoS_2_ nanoflowers on the graphene surface result in thinner MoS_2_ layers, contributing to this shift and further enhancing the catalytic activity for polysulfides.

Figure 3b,c shows the N_2_ adsorption–desorption isotherms and pore size distribution curves of MoS_2_, MoS_2_-G, and MoS_2_-NG. The calculated specific surface area of MoS_2_-NG is the largest at 59.48 cm^2^ g^−1^, followed by MoS_2_-G (16.87 cm^2^ g^−1^) and MoS_2_ with the smallest specific surface area of 7.05 cm^2^ g^−1^. The introduction of GO significantly alleviates the aggregation of MoS_2_ nanoflowers, and the PANI modification further promotes the uniform distribution of MoS_2_ nanoflowers on GO, thereby greatly increasing the specific surface area of the material. This enhancement in specific surface area is beneficial for the adsorption and catalytic conversion of polysulfides. All three materials exhibit mesoporous structures, with the pore sizes of MoS_2_ primarily centered around 29 nm. For MoS_2_-G and MoS_2_-NG, most pore diameters are also around 28 nm, but some pores are as small as 4 nm. This suggests that the pore size of MoS_2_ nanoflowers ranges from 25 to 30 nm, while the pore size of GO is around 4 nm.

To further verify the chemical composition and elemental states of MoS_2_-NG, X-ray photoelectron spectroscopy (XPS) was conducted and analyzed. The XPS survey spectrum (Figure 3d) indicates the presence of C, O, N, Mo, and S elements in MoS_2_-NG, with atomic percentages of 72.52%, 13.48%, 5.35%, 2.62%, and 6.03%, respectively. The high-resolution C 1s spectrum (Figure 3e) can be deconvoluted into six peaks, corresponding to different carbon-containing functional groups: C–C/C=C (284.6 eV), C–N (285.5 eV), C–O (285.7 eV), C=N (286.3 eV), C=O (286.8 eV), and O–C=O (288.8 eV). The O 1s spectrum (Figure 3f) can be divided into three peaks, attributed to the oxygen-containing functional groups on the graphene surface, including C=O (530.7 eV), C–OH (531.6 eV), and COOH (532.8 eV).

The high-resolution N 1s spectrum (Figure 3g) is fitted into four peaks, corresponding to Mo–N bonds, pyridinic nitrogen, pyrrolic nitrogen, and graphitic nitrogen, with binding energies at 395.4 eV, 398.1 eV, 399.8 eV, and 400.2 eV [34], respectively. This nitrogen doping is beneficial for enhancing the catalytic performance of MoS_2_, as the shorter Li–S bond length and lower interfacial formation energy in N-doped MoS_2_ facilitate the nucleation and growth of Li_2_S compared to pure MoS_2_ [29]. In the Mo 3d spectrum (Figure 3h), the peaks at 229.5 eV and 232.8 eV correspond to Mo^4+^ 3d_5/2_ and Mo^4+^ 3d_3/2_, while the peaks at 232.6 eV and 236.0 eV represent Mo^6+^ 3d_5/2_ and Mo^6+^ 3d_3/2_. Additionally, the peak at 226.8 eV is assigned to the S 2s orbital [35]. The high-resolution S 2p spectrum (Figure 3i) shows peaks at 162.3 eV and 163.2 eV, corresponding to terminal S^2−^ and bridging S_2_^2−^, respectively.

### 2.2. Catalytic Conversion Kinetics of MoS_2_-NG for Polysulfides

**Figure 4:**
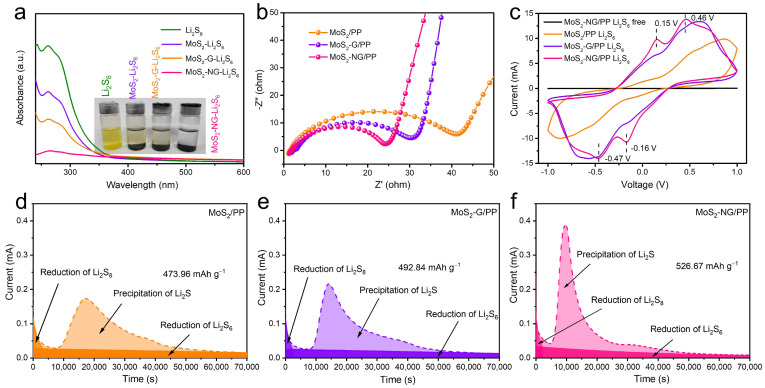
(**a**) UV-vis spectra of blank Li_2_S_6_ solution and digital photo of Li_2_S_6_ solution adsorbed by MoS_2_, MoS_2_-G, and MoS_2_-NG, respectively. Nyquist plots (**b**) and symmetric cell CV curves (**c**) of the cell assembled with MoS_2_/PP, MoS_2_-G/PP, and MoS_2_-NG/PP separators. Potentiostatic discharge curves of Li_2_S_8_ on the surface of MoS_2_/PP (**d**), MoS_2_-G/PP (**e**), and MoS_2_-NG/PP (**f**) separators at 2.05 V.

The chemical adsorption of polysulfides is a crucial prerequisite for materials to effectively catalyze their conversion. To evaluate the adsorption capacity of MoS_2_, MoS_2_-G, and MoS_2_-NG for polysulfides, we conducted an experiment using Li_2_S_6_ as a model compound. Equal volumes and concentrations of Li_2_S_6_ solution were added to equal masses of MoS_2_, MoS_2_-G, and MoS_2_-NG, followed by shaking for adsorption. Figure 4a presents digital photographs of the Li_2_S_6_ solutions after 12 h of rest. The initial Li_2_S_6_ solution was yellow, and after adsorption with the different materials, the solution color lightened to varying degrees, indicating that all three materials have some capacity for chemical adsorption of polysulfides. Notably, the solution with MoS_2_-NG became almost colorless, suggesting that Li_2_S_6_ was completely adsorbed, while the solutions with MoS_2_ and MoS_2_-G remained slightly yellow. This visual comparison indicates that MoS_2_-NG has the strongest adsorption capacity for polysulfides, followed by MoS_2_-G and MoS_2_.

To further characterize the concentration of Li_2_S_6_ in the supernatant, we measured the UV-vis absorption spectra. The characteristic absorption peak of Li_2_S_6_ is observed between 250 and 350 nm. According to the Lambert–Beer law, the concentration of the solution is proportional to its absorbance. Comparing the absorbance values of the three solutions, we found that the concentration of Li_2_S_6_ after adsorption was lowest for MoS_2_-NG, followed by MoS_2_-G and MoS_2_. This confirms that MoS_2_-NG has superior chemical adsorption capacity for polysulfides, surpassing both MoS_2_-G and MoS_2_. The enhanced adsorption capacity of MoS_2_-NG can be attributed to the PANI modification, which increases the adsorption sites on GO for polysulfides and ensures a more uniform distribution of MoS_2_ on its surface without aggregation. The expanded interlayer spacing of MoS_2_ also exposes more in-plane active sites, further strengthening its adsorption capacity for polysulfides.

We also conducted electrochemical impedance spectroscopy (EIS) tests on cells assembled with different separators under open-circuit voltage, as shown in Figure 4b. In the Nyquist plot, the diameter of the semicircle corresponds to the charge transfer resistance. Due to the poor conductivity of pure MoS_2_, the introduction of GO improves the overall conductivity of the material and provides pathways for lithium-ion transport, which plays a crucial role in optimizing redox kinetics. Among the samples, the MoS_2_-NG/PP separator exhibited the smallest semicircle, indicating the most significant catalytic effect on polysulfides. This finding is further supported by the results from the symmetric cell tests. As shown in Figure 4c, the cyclic voltammetry (CV) curves of the symmetric cells assembled with MoS_2_-NG/PP separator exhibit four distinct peaks at 0.15 V/−0.47 V and −0.16 V/0.46 V. The peaks at lower potentials with higher current responses indicate that MoS_2_-NG has a stronger catalytic effect on polysulfides compared to MoS_2_ and MoS_2_-G, which show peaks at higher potentials with lower current responses.

Additionally, to further confirm that MoS_2_-NG can more effectively promote the nucleation and growth of Li_2_S, we conducted potentiostatic discharge tests at 2.05 V using MoS_2_/PP, MoS_2_-G/PP, and MoS_2_-NG/PP as separators, carbon fiber felt as the current collector, and Li_2_S_8_ as the active material. The discharge curves are shown in Figure 4d–f. A comparative analysis reveals that the peak current appears earliest with the MoS_2_-NG/PP separator, followed by MoS_2_-G/PP, and lastly with the MoS_2_/PP separator. Moreover, the area under the peak, calculated according to Faraday’s law and corresponding to the capacity released by Li_2_S deposition, indicates that the specific capacity is significantly higher for the MoS_2_-NG/PP separator (526.67 mAh g^−1^) compared to the MoS_2_-G/PP (492.84 mAh g^−1^) and MoS_2_/PP (473.96 mAh g^−1^) separators. These results collectively demonstrate that MoS_2_-NG exhibits higher activity compared to MoS_2_ and MoS_2_-G. It not only optimizes the redox kinetics of the battery system but also significantly enhances the catalytic conversion of polysulfides, leading to more effective Li_2_S nucleation and growth.

### 2.3. Electrochemical Performance of MoS_2_-NG/PP Separator in Lithium–Sulfur Batteries

**Figure 5:**
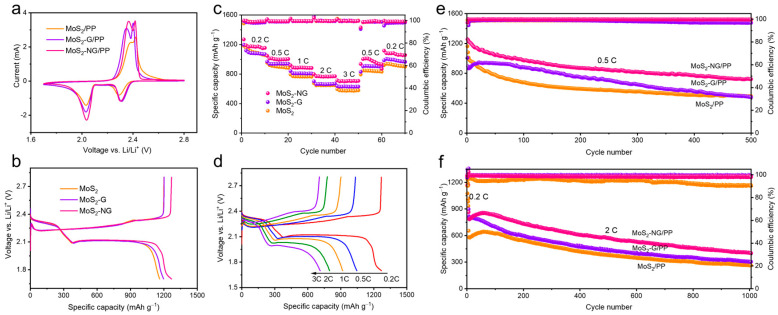
CV curves at a scan rate of 0.1 mV s^−1^ (**a**) and galvanostatic discharge/charge curves at a current density of 0.2 C (**b**) of cells assembled based on MoS_2_/PP, MoS_2_-G/PP, and MoS_2_-NG/PP separators. (**c**) Rate performance of cells with different separators. (**d**) Galvanostatic discharge/charge curves of the MoS_2_-NG/PP separator at various rates. Long-term cycling performance of cells with MoS_2_/PP, MoS_2_-G/PP, and MoS_2_-NG/PP separators at 0.5 C (**e**) and 2 C (**f**).

To demonstrate the enhancement of the overall electrochemical performance of lithium–sulfur batteries, MoS_2_-NG/PP separators were used to assemble coin cells, which were then subjected to cyclic voltammetry (CV), long-cycle charge/discharge, and rate performance tests. Figure 5a shows the CV curves of cells assembled with MoS_2_/PP, MoS_2_-G/PP, and MoS_2_-NG/PP separators within a voltage range of 1.7–2.8 V. Two distinct reduction peaks appear at approximately 2.3 V and 2.0 V, corresponding to the reduction of solid S_8_ to soluble polysulfides and their further reduction to the final solid product Li_2_S, respectively. The two oxidation peaks correspond to the re-oxidation of Li_2_S into polysulfides and further oxidation to S_8_. Comparing the cathodic scan curves of the three separators, the first reduction peak of MoS_2_-G/PP and MoS_2_-NG/PP shows a similar peak current and position, while MoS_2_/PP exhibits a more delayed peak with a lower peak current. Although the second reduction peak positions are close for all three separators, MoS_2_-NG/PP exhibits the highest current response, indicating a superior catalytic effect of MoS_2_-NG in facilitating the conversion of polysulfides to Li_2_S, consistent with the previous Li_2_S deposition experiments. During the anodic scan, MoS_2_-NG also demonstrates the highest peak current, suggesting that it optimizes the redox reaction kinetics within the battery system.

Figure 5b displays the constant current charge/discharge curves of cells with MoS_2_/PP, MoS_2_-G/PP, and MoS_2_-NG/PP separators at 0.2 C. The discharge process shows two distinct voltage plateaus: the first plateau around 2.35 V corresponds to the ring-opening reaction of S_8_ converting to polysulfides, and the second plateau around 2.1 V corresponds to the further conversion of polysulfides to Li_2_S, theoretically contributing 75% of the total discharge capacity. The charge curve indicates the dissolution and conversion of Li_2_S back into polysulfides and their re-oxidation to S_8_. After the first discharge plateau, the specific capacities of MoS_2_/PP, MoS_2_-G/PP, and MoS_2_-NG/PP separators differ only slightly, all around 380 mAh g^−1^. However, during the second discharge stage, corresponding to the conversion of polysulfides to Li_2_S, MoS_2_-NG/PP exhibits a longer second discharge plateau, releasing a more specific capacity. This is attributed to its strong catalytic effect, which effectively induces the nucleation and growth of Li_2_S, thereby releasing more capacity in this stage.

Figure 5c presents the rate performance of cells with the three different separators, with charge/discharge rates ranging from 0.2 C to 3 C and finally back to 0.2 C. The cell with the MoS_2_-NG/PP separator delivers specific capacities of 1269, 1047, 918, 798, and 714 mAh g^−1^ at 0.2, 0.5, 1, 2, and 3 C, respectively. When the rate returns to 0.5 and 0.2 C, the specific capacities recover to 1004 and 1117 mAh g^−1^, respectively. In contrast, due to the relatively poor chemical adsorption and catalytic performance of MoS_2_ and MoS_2_-G towards polysulfides, the discharge capacities of cells with MoS_2_/PP and MoS_2_-G/PP separators are consistently lower than those with MoS_2_-NG/PP separators at various current densities. When compared to other MoS_2_-based materials reported in the literature [30,36,37,38], MoS_2_-NG exhibits significantly higher rate performance and specific capacities. This demonstrates that MoS_2_-NG has excellent catalytic activity, effectively suppressing the shuttle effect of polysulfides and inducing the nucleation and growth of Li_2_S, thereby enhancing the rate performance of the battery. Figure 5d shows the constant current charge/discharge curves of the cell with the MoS_2_-NG/PP separator at different rates. As the current density increases, only minimal polarization is observed, and even at 3 C, the two distinct charge/discharge plateaus are well maintained, indicating that MoS_2_-NG plays a crucial role in optimizing the reaction kinetics of the battery. The voltage drop across the resistance, as expected from Ohm’s law (the product of current and resistance), was minimized, which may be attributed to the N-doping effect that improves conductivity and reduces charge transfer resistance. This further supports the role of heteroatom doping in enhancing the overall capacity performance by minimizing internal resistance [39].

In addition to rate performance, long-cycle stability is also a critical indicator of the electrochemical performance of lithium–sulfur batteries. Therefore, we conducted long-cycle charge/discharge tests at 0.5, 1, and 2 C. As shown in Figure 5e, the initial specific capacities at 0.5 C for cells with MoS_2_-NG/PP, MoS_2_-G/PP, and MoS_2_/PP separators were 1255, 1006, and 1161 mAh g^−1^, respectively. After 500 cycles, the specific capacities were maintained at 722, 482, and 494 mAh g^−1^, with capacity decay rates of 0.085%, 0.104%, and 0.115% per cycle, respectively. Similarly, at 1 C (Figure S5) and 2 C (Figure 5f), cells with the MoS_2_-NG/PP separator exhibited higher specific capacities and lower capacity decay, indicating that MoS_2_-NG effectively catalyzes polysulfides and promotes the formation of Li_2_S, thereby suppressing the shuttle effect and enhancing the specific capacity and cycling stability of the battery. Notably, the Coulombic efficiency of the cell with the MoS_2_/PP separator sharply declined with continued cycling, likely due to the diminished ability of MoS_2_ to adsorb and catalyze polysulfides after prolonged cycling, leading to an exacerbated shuttle effect and reduced Coulombic efficiency.

## 3. Materials and Methods

### 3.1. Material Preparation

Synthesis of PANI-GO: To prepare PANI-GO, 80 mg of GO (purchased from GaoxiTech Company, Hangzhou, China) was dispersed in 40 mL of deionized water and sonicated for 2 h to form a uniform GO dispersion. Separately, 1.2 g of aniline (C_6_H_7_N, 99.5%, Shanghai Macklin Biochemical Co., Ltd., Shanghai, China) was dissolved in 40 mL of dilute hydrochloric acid (1 mol L^−1^, AR, Sinopharm Chemical Reagent Co., Ltd., Shanghai, China) and then added to the GO dispersion, followed by stirring for 30 min. Subsequently, 0.8 mL of 30% hydrogen peroxide solution (H_2_O_2_, AR, Sinopharm Chemical Reagent Co., Ltd., Shanghai, China) solution was added dropwise to the mixture, which was stirred at room temperature for 24 h, resulting in a dark green dispersion. The product was centrifuged, washed with deionized water until neutral, and then redispersed in 80 mL of deionized water to obtain a PANI-GO dispersion with a concentration of 1 mg mL^−1^.

The synthesis of MoS_2_-NG, MoS_2_-G, and MoS_2_ was carried out through a hydrothermal method followed by calcination. For MoS_2_-NG, 800 mg of ammonium molybdate tetrahydrate ((NH_4_)_6_Mo_7_O_24_·4H_2_O, 99%, Shanghai Macklin Biochemical Co., Ltd., Shanghai, China), 2 g of thiourea (CH_4_N_2_S, AR, Sinopharm Chemical Reagent Co., Ltd., Shanghai, China), and 200 mg of polyvinylpyrrolidone (PVP, K30, Shanghai Dibai Biotechnology Co., Ltd., Shanghai, China) were added to 80 mL of PANI-GO dispersion, stirred for 2 h at room temperature, and then transferred to a hydrothermal reactor at 200 °C for 20 h. The product was centrifuged, washed with deionized water and ethanol, freeze-dried, and finally calcined at 800 °C under a nitrogen atmosphere at a heating rate of 10 °C per minute to obtain MoS_2_-NG. The MoS_2_ content in the composite was approximately 40.7% (Figure S6). MoS_2_-G was synthesized similarly, using a GO dispersion instead of PANI-GO, while MoS_2_ was prepared without any GO or PANI-GO, following the same procedure.

### 3.2. Material Characterization

The microstructure and elemental composition of the samples were analyzed using field emission scanning electron microscopy (SEM, JEOL JEM7800F, Peabody, MA, USA) and transmission electron microscopy (TEM, JEOL JEM-2100). The crystalline structure of the samples was examined through powder X-ray diffraction (XRD) using an X-ray diffractometer (Rigaku Miniflex, Rigaku Corporation, Tokyo, Japan) with Cu Kα radiation (λ = 0.15406 nm) over a diffraction angle (2θ) range of 5° to 80°. The Raman spectra of the samples were obtained using a Raman spectrometer (LabRAM Aramis, HORIBA Scientific, Palaiseau, France) to characterize the degree of graphitization and the presence of MoS_2_. The N_2_ adsorption–desorption isotherms were measured using a Micromeritics ASAP 2020 Plus analyzer (Norcross, GA, USA) for specific surface area (BET) and pore size distribution (BJH) analysis. X-ray photoelectron spectroscopy (XPS) of the samples was performed using a Thermo Escalab 250 spectrometer (Thermo Fisher Scientific, Waltham, MA, USA) with an Al Kα source (hν = 1486.6 eV), and the full spectrum as well as the XPS spectra of individual elements were calibrated using the C 1s peak at 284.6 eV. The ultraviolet–visible (UV-vis) absorption spectra of Li_2_S_6_ in the polysulfide adsorption experiments were measured using a PerkinElmer Lambda 6500s spectrophotometer (PerkinElmer, Waltham, MA, USA).

### 3.3. Li_2_S_6_ Adsorption Experiment

Li_2_S_6_ solution was prepared by dissolving sulfur (S) and lithium sulfide (Li_2_S) in a molar ratio of 5:1 in DME, followed by stirring at room temperature for 24 h in a sealed glovebox. Then, 20 mg of MoS_2_-NG, MoS_2_-G, and MoS_2_ were each placed into transparent glass vials, and an equal amount of Li_2_S_6_ solution was added to each. After shaking and allowing the mixtures to sit for 12 h, the color of the supernatant was observed and the concentration of Li_2_S_6_ was further analyzed using UV–visible absorption spectroscopy.

### 3.4. Li_2_S_6_ Symmetric Cell Assembly and Testing

A Li_2_S_6_ electrolyte solution (0.1 M) was prepared by dissolving S and Li_2_S in a molar ratio of 5:1 in lithium–sulfur electrolyte (1 M LiTFSI dissolved in a 1:1 volume ratio of DOL/DME, with 1 wt% LiNO_3_) and stirring at room temperature for 24 h in a glovebox. Carbon paper with a diameter of 12 mm was used as both the working electrode and the counter electrode. Two MoS_2_-NG/PP, MoS_2_-G/PP, and MoS_2_/PP separators were placed with the modified layers facing the working and counter electrodes, and 25 μL of Li_2_S_6_ electrolyte was added to each side to assemble symmetric cells. Cyclic voltammetry (CV) tests were conducted with a voltage range of −1 to 1 V at a scan rate of 10 mV s^−1^ to record the current–voltage curves.

### 3.5. Li_2_S Nucleation Experiment

Li_2_S_8_ electrolyte solution (0.2 M) was prepared by dissolving S and Li_2_S in a molar ratio of 7:1 in lithium–air electrolyte (1 M LiTFSI dissolved in tetraethylene glycol dimethyl ether, with 1 wt% LiNO_3_) and stirring at room temperature for 24 h in a glovebox. Carbon fiber felt was used as the cathode, lithium foil as the anode, and MoS_2_-NG, MoS_2_-G, and MoS_2_/PP were used as separators. The cathode side and anode side were each treated with 25 μL of Li_2_S_8_ electrolyte and 20 μL of lithium–air electrolyte, respectively, to assemble coin cells. The cells were discharged at a constant current of 0.112 mA to 2.06 V, followed by potentiostatic discharge at 2.05 V to allow Li_2_S nucleation and growth until the current dropped below 10^−5^ A. The rate of Li_2_S nucleation growth was calculated using Faraday’s law.

### 3.6. Cell Assembly and Electrochemical Testing

CNTs and sublimed sulfur were mixed in an 8:2 mass ratio and ground together, then reacted at 155 °C for 12 h under an Ar atmosphere in a tube furnace to obtain the CNTs/S composite. A cathode slurry was prepared by ball-milling S/CNTs, Super P, and LA133 in a 7:2:1 mass ratio, with water and ethanol as solvents. The resulting slurry was coated onto an aluminum foil current collector using a coating machine, with the coating thickness adjusted to 10 μm and an active material loading of 1 mg cm^−2^. After drying overnight at 60 °C, the CNTs/S electrodes were cut into 12 mm diameter disks for use.

Using CNTs/S as the cathode, MoS_2_-NG/PP, MoS_2_-G/PP, and MoS_2_/PP as separators, lithium foil as the anode, and a 1 M LiTFSI solution dissolved in a 1:1 volume ratio of DOL/DME with 2 wt% LiNO_3_ as the electrolyte, CR2032-type coin cells were assembled in an argon-filled glovebox and tested for electrochemical performance. Constant current charge/discharge tests and rate performance tests were conducted using a LAND CT2001A battery tester (Landt Instruments, Vestal, NY, USA) with a voltage window of 1.7 to 2.8 V. The current density and specific capacity were calculated based on the sulfur content in the cathode (1 C = 1675 mAh g^−1^). CV and electrochemical impedance spectroscopy (EIS) were performed using an Autolab electrochemical workstation (Metrohm, Herisau, Switzerland). The CV potential window was set to 1.7 to 2.8 V, with a scan rate of 0.1 to 0.5 mV s^−1^, and the EIS frequency range was 0.01 to 100 kHz, with a perturbation voltage of 5 mV.

## 4. Conclusions

In this work, PANI was employed to modify the surface charge of GO, creating sites capable of uniformly adsorbing Mo_7_O_24_^6−^, which ensured the homogeneous growth of MoS_2_ nanoflowers on the GO surface without aggregation. The resulting MoS_2_-NG was used as a modification material for the separator in lithium–sulfur batteries. Electrochemical performance tests demonstrated that even at a high rate of 3 C, the battery with the MoS_2_-NG/PP separator delivered a specific capacity of 714 mAh g^−1^. Moreover, after 500 cycles at 0.5 C, the capacity decay rate per cycle was only 0.085%, indicating that MoS_2_-NG effectively suppressed the shuttle effect of polysulfides and facilitated the growth of Li_2_S, thereby enhancing both the long-term cycling stability and rate performance of the battery. The layered structure of GO contributed to the physical confinement of polysulfides, while the nitrogen-doped sites on the PANI-modified GO surface, in synergy with the polar surface of MoS_2_, provided chemical adsorption of polysulfides. Crucially, the uniform growth of MoS_2_ on the GO surface prevented the masking of catalytic sites due to excessive stacking. The increased interlayer spacing of MoS_2_ further exposed more in-plane active sites, promoting the catalytic conversion of polysulfides during charge/discharge processes and inducing the nucleation and growth of Li_2_S, thereby optimizing the kinetics of the battery reactions.

## Data Availability

Data are contained within the article and Supplementary Materials.

## Supplementary Materials

The following supporting information can be downloaded at: https://www.mdpi.com/article/10.3390/molecules29204968/s1, Figure S1: SEM images of GO (a–c) and PANI-GO (d–f) at different magnifications; (g–i) Element distribution diagram of PANI-GO (C, N, O elements); Figure S2: Zeta potentials of GO (a) and PANI-GO (b); Figure S3: SEM images of top-surface and cross section of PP (a–c), MoS_2_/PP (d–f), MoS_2_-G/PP (g–i) and MoS_2_-NG/PP (j–l) separators; Figure S4: Raman spectra of MoS_2_, MoS_2_-G and MoS_2_-NG; Figure S5: Long-term cycling performance of cells with MoS_2_/PP, MoS_2_-G/PP, MoS_2_-NG/PP separators at 1 C; Figure S6: EDS spectrum showing the elemental composition of the MoS_2_-NG composite.
