# Supplementary material for: Nitrogen-Doped Graphene Uniformly Loaded with Large Interlayer Spacing MoS2 Nanoflowers for Enhanced Lithium–Sulfur Battery Performance
Source: Molecules. 2024 Oct 21;29(20):4968. doi: 10.3390/molecules29204968 (PMC11509959; doi:10.3390/molecules29204968)
Supplement: Supplementary file 1 [file molecules-29-04968-s001.zip › molecules-3211463-supplementary.pdf]

Nitrogen-Doped Graphene Uniformly Loaded with Large Interlayer Spacing MoS2 Nanoflowers for Enhanced Lithium–Sulfur Battery Performance

Zhen Wu [1,2,3,*,†], Wenfeng He [2,†], Renjie Xie [1], Xuan Xiong [1], Zihan Wang [1], Lei Zhou [1], Fen Qiao [1], Junfeng Wang [1], Yan Zhou [1], Xinlei Wang [1], Jiajia Yuan [2], Tian Tang [2], Chenyao Hu [4], Wei Tong [5], Lubin Ni [6], Xin Wang [2] and Yongsheng Fu [2,*]

1 School of Energy and Power Engineering, Jiangsu University, Zhenjiang 212013, China; 18719810161@163.com (R.X.); 19709101790@163.com (X.X.); l.zhou@ujs.edu.cn (L.Z.); wangxinlei@ujs.edu.cn (X.W.)

2 Key Laboratory for Soft Chemistry and Functional Materials of Ministry of Education, Nanjing University of Science and Technology, Nanjing 210094, China; jiajiayuan@njust.edu.cn (J.Y.)

3 FEB Research Institute, Far East Battery, Wuxi 214200, China

4 College of Science & Institute of Materials Physics and Chemistry, Nanjing Forestry University, Nanjing 210037, China

5 School of Mechanical and Aerospace Engineering, Nanyang Technological University, 50 Nanyang Avenue, Singapore 639798, Singapore; tomson90@126.com

6 School of Chemistry and Chemical Engineering, Yangzhou University, Yangzhou 225002, China; lbni@yzu.edu.cn

* Correspondence: zhenwu@ujs.edu.cn (Z.W.); fuyongsheng@njust.edu.cn (Y.F.)

† These authors contributed equally to this work.

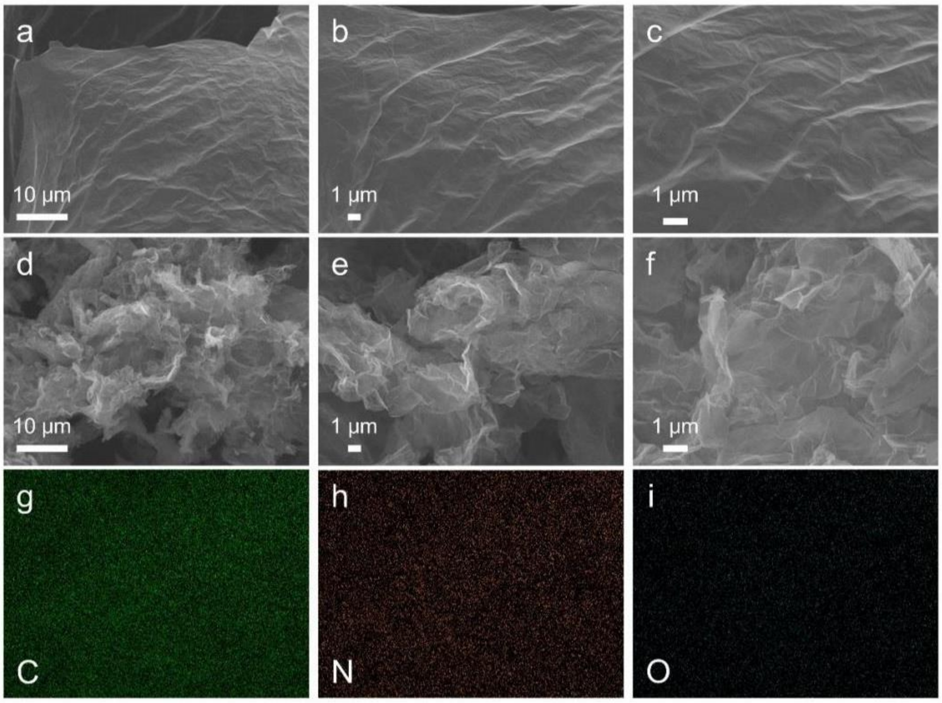

Figure S1. SEM images of GO (a-c) and PANI-GO (d-f) at different magnifications; (g-i) Element distribution diagram of PANI-GO (C, N, O elements).

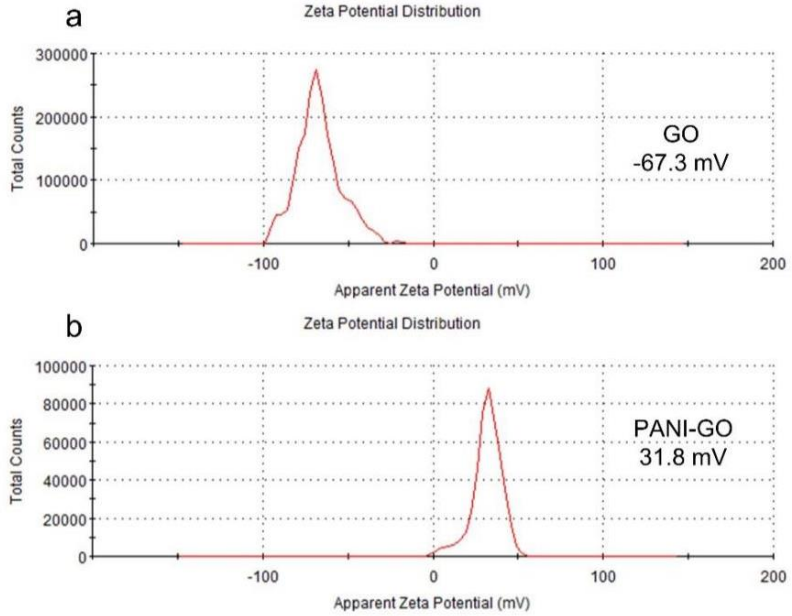

Figure S2. Zeta potentials of GO (a) and PANI-GO (b).

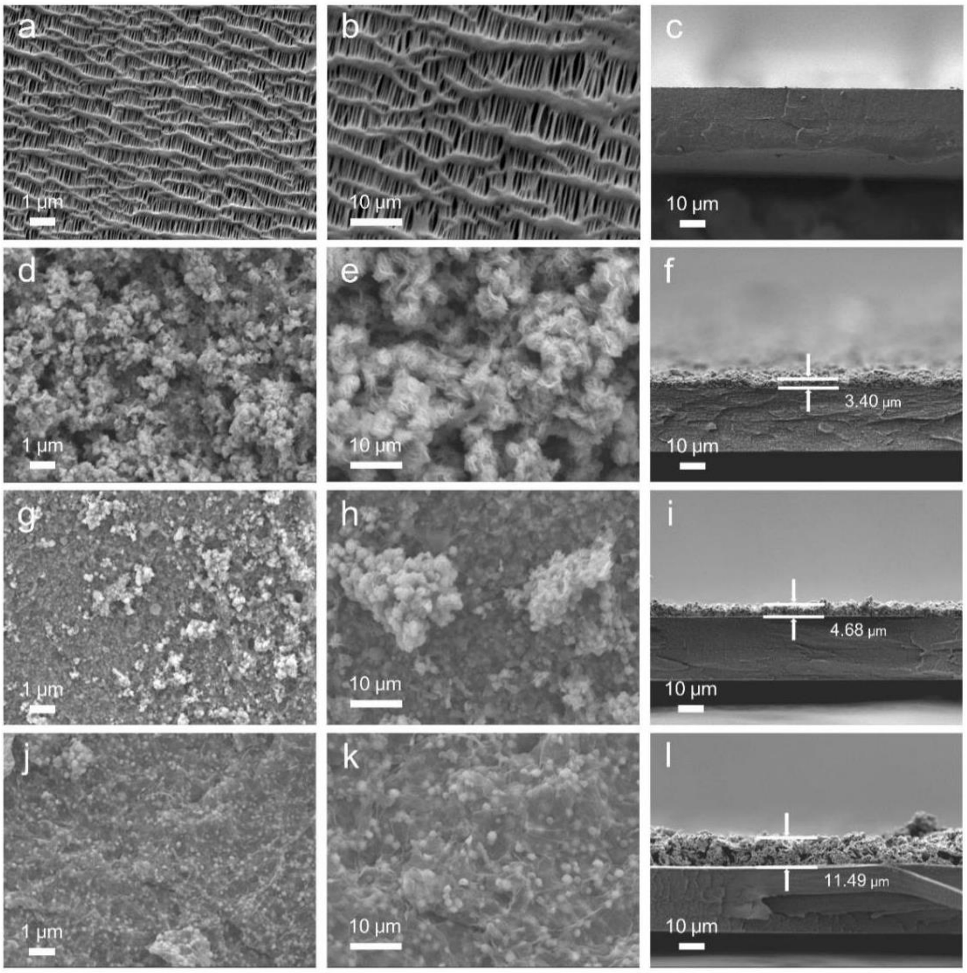

Figure S3. SEM images of top-surface and cross section of PP (a-c), MoS_2/PP (d-f), MoS_2-G/PP (g-i) and MoS_2-NG/PP (j-l) separators.

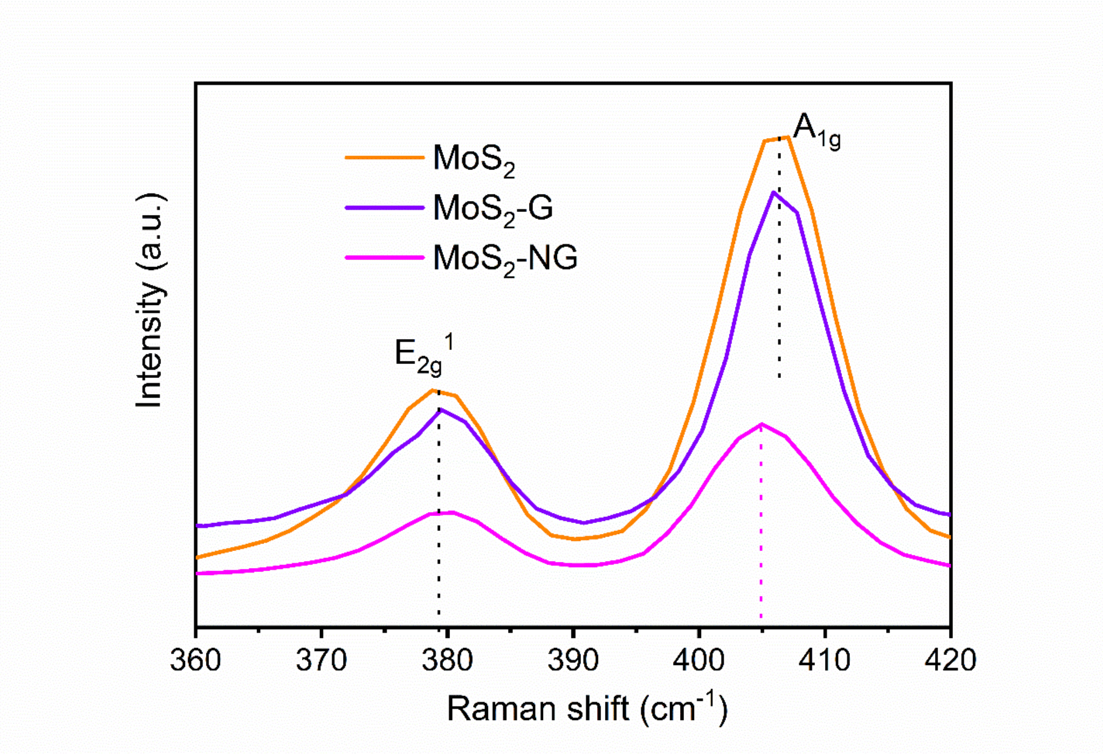

Figure S4. Raman spectra of MoS_2, MoS_2-G and MoS_2-NG.

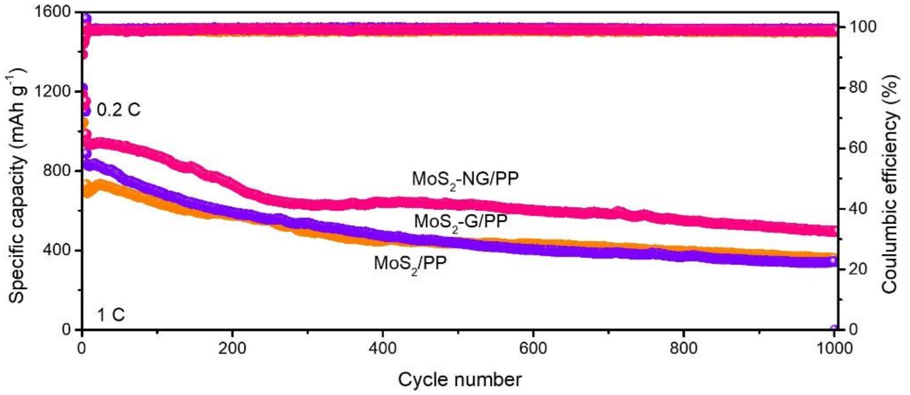

Figure S5. Long-term cycling performance of cells with MoS_2/PP, MoS_2-G/PP, MoS_2-NG/PP separators at 1 C.

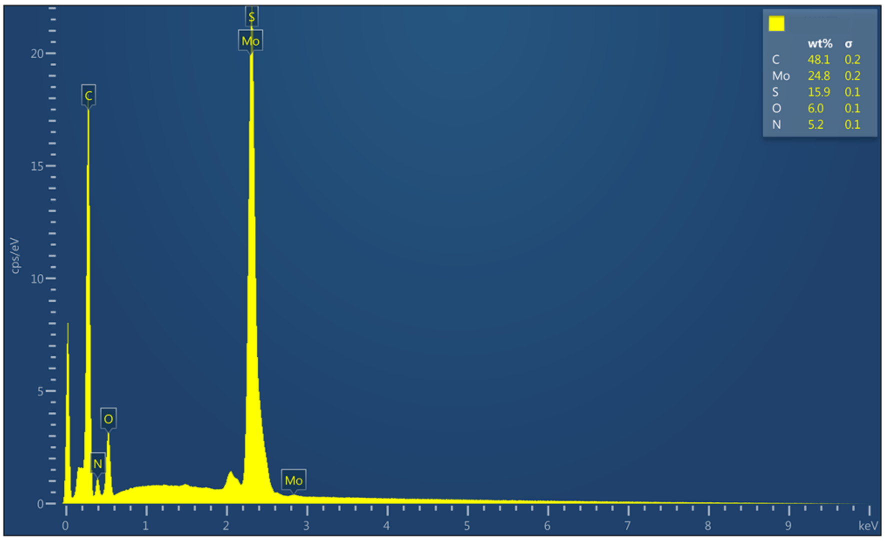

Figure S6. EDS spectrum showing the elemental composition of the MoS_2-NG composite.
